# Supplementary figures and images for: A cross-sectional study on the effects of bedtime administration of selective α1 adrenoceptor antagonists on nocturnal blood pressure in elderly patients with benign prostate hyperplasia
Source: PeerJ. 2025 Apr 1;13:e19165. doi: 10.7717/peerj.19165 (PMC11970415; doi:10.7717/peerj.19165)

## Flow Diagram

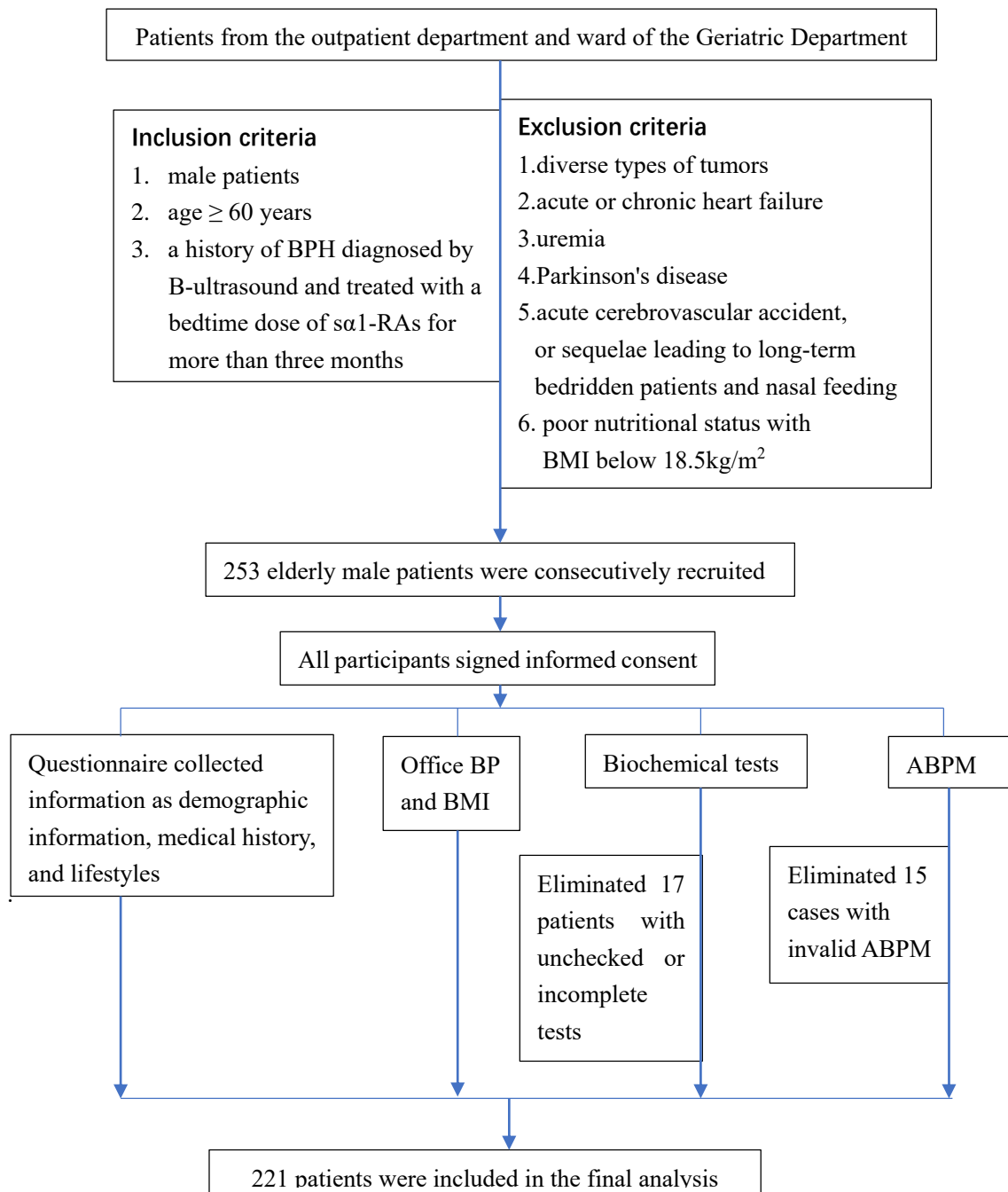

Supplement: Supplemental Information 2 [file peerj-13-19165-s002.pdf]
