# Supplementary material for: A cross-sectional study on the effects of bedtime administration of selective α1 adrenoceptor antagonists on nocturnal blood pressure in elderly patients with benign prostate hyperplasia
Source: PeerJ. 2025 Apr 1;13:e19165. doi: 10.7717/peerj.19165 (PMC11970415; doi:10.7717/peerj.19165)
Supplement: Supplemental Information 3 [file peerj-13-19165-s003.doc]

**研究方案**

**老年夜间血压异常与靶器官功能的关系**

**版本号：4.0**

**版本日期：2021年3月31日**

**主要研究者：陈朝婷**

**联系地址：上海市第九人民医院老年科**

**上海市制造局路639号，上海200011**

**电话：+86 13301993917**

**传真：+86 21 2327 1699**

**电子邮箱：cctviewer@163.com**

**一、研究背景**

高血压是老年人常见的慢性病，我国高血压发病率随着年龄增长呈现增高趋势，60岁以上的男性和女性分别为41.6%和38.4%。高血压是心血管疾病的主要危险因素，也是位居全球的首位死因。我国高血压的心脑血管风险随着年龄的增长而增加，因此重视老年高血压诊治有利于显著降低个人和社会的医疗负担。高血压防治的根本目标是降低血压对靶器官的损害，减少心脑血管并发症和死亡总风险。近年随着动态血压的广泛使用，国内外研究发现亚裔比欧洲裔普遍存在夜间血压增高现象，仅孤立性夜间高血压在我国的患病率就达到10.9%，而西方国家仅6%。研究发现夜间高血压的心脑血管风险明显高于日间高血压，与多种靶器官损伤密切相关，是独立于24小时平均血压的恶性心血管终点事件的预测因子。但夜间高血压往往依靠动态血压监测发现，因而是高血压临床诊治的盲区，属于隐匿性高血压的范畴。在经治疗的高血压患者中夜间高血压较日间高血压与致死和非致死性心血管事件（包括脑卒中、心肌梗死和心血管死亡）更密切相关。夜间高血压常见于老年人，与糖尿病、慢性肾病和阻塞性睡眠呼吸暂停3种老年常见病密切相伴，并与失眠症、认知功能障碍、虚弱、行走缓速、心力衰竭等老年疾患相关。夜间血压增高与机体神经调节机制异常相关,可继发于交感神经系统活性增高。也有研究发现夜间高血压与动脉硬化和高盐饮食有关。当前高血压管理的方向是及早、全天候控制血压，临床医师不仅要关注老年高血压患者诊室血压达标,更要重视夜间高血压的早期诊治，清除防控盲区，从而有效提升我国高血压的知晓率、治疗率和控制率,将靶器官损害和心血管风险降到最低。

同时，由于全身机能老化、压力感受器敏感性降低、血管结构和舒缩功能改变以及机体衰弱、多病共存、多药联用等各种因素，即便是高血压患者，在老年人其夜间血压也存在另一种倾向即夜间低血压。国内外研究发现，短期低血压可导致晕厥、跌倒、急性心脑血管事件发生甚至猝死。糖尿病、帕金森病和高血压的患者，直立性低血压的发生率明显增加，分别达到58%、28%和32%;在急诊病房和老年护理院，直立性低血压的发病率分别可高达41-50%。这使老年人起夜时由于低血压导致的跌倒意外和各种心脑血管的风险增加。Framingham一项对75岁以上的老人进行为期38年的研究发现，在合并有心血管疾病的老人其收缩压与心血管死亡率呈U型曲线，当收缩压低于120mmHg时，无论性别其心血管死亡率均明显增加。而高血压老年患者长期服用的降压药本身以及因多种疾病情况联合使用的扩血管药、镇静催眠药、抗抑郁药等也会引发慢性的夜间低血压，导致全身脏器因长期低灌注出现器官衰退加速甚至衰竭死亡。

无论是夜间高血压还是低血压，都会对老年人靶器官的功能产生严重的影响并增加心脑血管风险。基于目前对老年人夜间血压异常状况和对靶器官功能影响、心脑血管事件风险全面评估的研究缺乏，因此，本研究拟通过动态血压监测对老年人夜间血压状况分组评估，明确夜间高血压和低血压的发生及相关影响因素，全面关注心、脑、肾和血管等靶器官受累程度并随访相关的心血管终点事件，将对老年高血压患者药物治疗方案的有效合理调整，避免因夜间血压过高、过低导致出血性或缺血性脑卒中、认知功能障碍或痴呆、慢性肾功能或心功能衰竭、急性心梗、晕厥、跌倒等恶性心脑血管事件，使老年人的全天候血压水平维持于有利于器官功能的理想灌注水平。

**二、研究目标**

本研究通过对老年高血压经治患者开展夜间血压异常的筛查和随访，（1）明确老年高血压经治患者夜间血压异常状况和相关危险因素，及与靶器官受损的相关性；（2）探寻涉及的神经内分泌、炎症、免疫机制对夜间血压异常及早诊治的生物标志物；（3）随访相关的心脑血管终点事件发生状况；从而对老年患者尤其是高龄老人安全有效的降压治疗方案和建立夜间血压异常可循的防治规范提供临床依据。

本研究紧扣高血压防治的根本目标——降低靶器官的损害和减少心脑血管并发症，关注心脑血管高危风险但目前研究有限的对象——老年高血压人群尤其是高龄老人，由于机体衰弱、机能减退、多病共存、多药联用等错综情况，着眼于夜间血压异常——这项对靶器官损害和心血管风险有显著影响但较隐匿易忽视的临床问题，不仅关注老年夜间高血压也重视夜间低血压对心血管风险的影响，综合分析夜间血压各种异常状况及影响因素，全面评估对重要靶器官的损害程度，探寻相关的神经内分泌、炎症、免疫标志物并随访相关的心脑血管终点事件发生。

**三、研究内容**

1.基线研究：

1）门诊和病房招募老年原发性高血压患者，在知情同意后根据入选和排除标准入组患者；

2）分组：根据动态血压监测夜间血压分3组：夜间高血压组、夜间低血压组、夜间正常血压组，三组各1000位患者完善流行病学资料收集、体格检查、实验室检查和各项特殊检查；

3）实验室检查：包括血尿常规、血生化（包括电解质、血脂、空腹血糖、糖化血红蛋白）、肾功能及尿微量白蛋白和尿蛋白/肌酐比值、凝血功能（包括纤维蛋白原、PT、APTT、TT）和D-二聚体、C反应蛋白（CRP）、同型半胱氨酸（Hcy）、RAAS因子（肾素、血尿AngII、血尿醛固酮）和免疫因子（TNF-α，IL-1β）检测；

4）特殊检查：动态血压、脑彩超（TCD）、心超、颈动脉B超、颅内外动脉成像（CTA/MRA）、头颅CT/MRI平扫及简易智能量表（MMSE）行认知功能测定；

2.随访研究：

1）随访指标和频率：对生存状况、疾病发生情况及血压，每半年进行一次随访；若无主要或次要终点事件发生，随访期5年；随访期满时复查生存状况、疾病发生情况及体格检查、与基线研究相同的实验室检查及特殊检查包括头颅MRI/CT平扫和脑血管MRA/CTA项目。

2）研究终点事件：主要终点：包括致死和非致死性缺血性脑卒中（ICD10编码: I63），不包括短暂脑缺血发作（TIA，G45）。次要终点：致死性和非致死性脑卒中（包括缺血性和出血性脑卒中及蛛网膜下腔出血, I60-64）；短暂脑缺血发作（G45）；全因死亡和心血管死亡（I00-I79）；致死和非致死性心血管病事件，包括心血管死亡（I00-I79）、冠心病事件（I20，I24-25，I46, R96, Z955）、心肌梗死 (I21-22)、心衰(I50)和肾衰(N17-19)等。

3.统计方法：单因素ANOVA检验及多重比较检验进行组间比较分析夜间血压异常的影响因素，多元线性逐步回归及logistic分析危险因素、ROC 曲线分析神经内分泌、炎症免疫标志物灵敏度和特异度。采用COX多元回归分析夜间血压异常（夜间高血压或夜间低血压）与各终点事件之间的关系。

**四、研究对象**

拟在上海第九人民医院老年科门诊及病房依次入选患者。

1.入选标准：年龄≥60岁，已明确原发性高血压且降压治疗≥1月以上，能长期随访者。

2.排除标准：

1. 确诊的继发性高血压患者；
2. 半年内的脑卒中（缺血性或出血性）患者；
3. 有2年内心肌梗死病史、房颤或频发早搏患者；
4. 其他心血管严重疾患（如心衰、肾衰、老年痴呆等）；
5. 肿瘤终末期；
6. 血管闭塞性坏疽；
7. 多发性动脉炎。

**五、观察内容**

1. 通过研究表格收集以下基本信息：
2. 人口学资料：患者姓名缩写、性别、民族、出生年月、文化程度等；
3. 疾病史：高血压病史、既往心血管疾病史、糖尿病史及高血压、心血管疾病和糖尿病的家族史等；
4. 个人史包括吸烟、饮酒、运动情况等。
5. 最近2周内服用降压药物、降脂药物、降糖药物、减肥药物、抗血小板或抗凝药物及其他药物情况；
6. 体格检查：测量身高、体重、腰围、臀围。腰围指腰部的最小周长，臀围指臀部的最大周长。用统一的电子血压计测量二次坐位非优势臂的上臂血压，同时记录心率。血压测量前需测量臂围，如果臂围超过32cm，则要用大袖带。
7. 实验室检查：
8. 血、尿常规；
9. 血生化：血电解质、血脂（总胆固醇、甘油三酯、低密度脂蛋白总胆固醇、高密度脂蛋白总胆固醇）、空腹血糖、糖化血红蛋白、肾功能（血清肌酐、尿素氮、肾小球滤过率、血清尿酸）；
10. 尿微量白蛋白：可以用晨尿或随机尿；
11. 测定微量白蛋白/肌酐比值；
12. 凝血机制（包括纤维蛋白原、PT、APTT、TT）和D-二聚体；
13. C反应蛋白（CRP）:乳胶免疫比浊法检测;
14. 同型半胱氨酸（Hcy）: 化学发光酶免疫法测定;
15. RAAS因子（肾素、血尿AngII、血尿醛固酮）检测；
16. 免疫因子（TNF-α，IL-1β）检测；
17. 24小时动态血压：使用经过认证的动态血压监测仪。白天（6点到22点）每20分钟，夜间（22点到6点）每30分钟测量一次血压。有效测量次数达80%以上，至少包含8次夜间血压为有效记录。
18. 经颅多普勒超声(TCD)；测量椎-基底动脉、大脑前、中、后动脉的收缩期及舒张期血流速度、搏动指数及阻力指数。
19. 心脏超声：测量左室后壁厚度(PWT)、室间隔厚度(IVST)、左室舒张末期内径(LVID)、左房内径、A峰、E峰等。测量左室后壁厚度、室间隔厚度时，需要包括心内膜厚度。使用改良的Devereux公式计算左室质量LVM(g)=0.8* 1.04*[(IVST+PWT+LVID)^3^-LVID^3^]+0.6。
20. 颈动脉超声：横断面和纵切面观察左右颈总、颈内、颈外动脉是否有斑块形成，记录斑块部位、大小及性质。选择颈总动脉分叉以下1.5cm处，测量颈动脉舒张末期内径及探头远端颈动脉内中膜厚度。取左右颈动脉的内中膜厚度的平均值用作分析。
21. 脑血管MRA/CTA检查；
22. 头颅MRI/CT平扫；
23. 认知功能检查：采用MMSE评分量表。

**五、样本量估计与统计分析**

样本量以前瞻性研究主要终点事件缺血性脑卒中的发生情况进行估计，缺血性脑卒中的年发病率为15/1000，3000例患者平均5年随访后，将会有225个事件，提供90%以上的研究效能。

研究数据将用SPSS24.0统计软件进行管理与统计分析。单因素ANOVA检验及多重比较检验进行组间比较分析夜间血压异常的影响因素，多元线性逐步回归及logistic分析危险因素、ROC 曲线分析神经内分泌、炎症免疫标志物灵敏度和特异度。采用COX多元回归分析夜间血压异常（夜间高血压或夜间低血压）与主要终点和次要终点之间的关系。

**六、时间安排**

2020年10月10日召开启动会，明确伦理的备案及研究方案的实施，2020年12月开始研究工作，2022年6月完成基线入选并开始随访， 2023年公布基线研究情况，2025年公布5年随访研究情况，2027年8月完成所有随访工作，2027年公开发表主要研究结果。

**七、组织实施**

本研究由上海交通大学医学院附属第九人民医院老年科启动实施，主要研究者陈朝婷全面负责课题的组织实施、质量控制、随访和统计分析工作等，老年科主任统筹协调，课题组成员在病例筛选评估、病史收集、标本送检、靶器官受损临床评估和病史资料收集、整理、随访和统计分析等方面按照分工合作的原则参加研究，明确各自的任务和时间节点，以保证课题顺利完成。上海市第九人民医院老年科门诊和病房具备完成本课题的人员和技术保证。本研究产出的成果（论文、奖项等），按贡献大小位列名次，共同申报和承担有关的科研课题。
